# The Future of Dentistry Electrically Considered

**Published:** 1896-08

**Authors:** G. H. Guy

**Affiliations:** New York


### THE FUTURE OF DENTISTRY ELECTRICALLY CONSIDERED.[1]

BY G. H. GUY,[2] NEW YORK.

Mr. President,—When the zealous chairman of your committee first asked me to respond to the toast of "The Future of Dentistry Electrically Considered," at your annual dinner, it seemed to me that he had come to the wrong man for his purpose, and, while I appreciated the honor very fully, I felt compelled to decline it. But he wrote me again, so adroitly and kindly, that I could hardly do anything else but consent. I am confronted by two difficulties. In the first place, I can tell you gentlemen little that you do not know already, and, in the second place, I can see before me, like a warning beacon, that wise old maxim, "Never prophesy unless you know."

Neither you nor I can say very much about what the mission of electricity in dentistry is going to be, but we know that it is destined to fill a high place,—I hope, I am sure, and I believe that it

---

[1] Read before the Central Dental Association of Northern New Jersey, April 20, 1896.

[2] Editor of the Electrical Review, New York City.

will satisfy the dentist as well as that of the public,—and that it is going to be comprehensive, pervasive, and beneficial, beyond any agency that has ever been known in the dental art.

As I propose to occupy but a very few minutes of your time, I shall confine myself to a brief reference to some of the departments of electrical work in which the members of the dental profession are likely to be deeply interested. In considering this question, it is a most hopeful sign that although until recently electricity has been but little employed in dental surgery, excepting for the driving of mechanisms, cauterization, the transillumination of soft tissue, or the illumination of cavities, yet the profession are taking kindly to it. A large section of your medical brethren are still regarding its progress and increasing popularity with distrust and blind unbelief. It is not long since, in calling on a leading fashionable physician in New York, I asked his opinion as to the likelihood of electricity being eventually taken up in earnest by the profession. He replied, "It will never be. I have tried it myself, and have come to the conclusion that there is nothing in it." The whole electrical apparatus of this physician turned out to be a small faradic battery, and he had been treating all ailments with the same kind of current. There are thousands of practitioners who still think as he did. But there are many signs which go to show that the progressive and intelligent section of the dental profession are advancing with the times, and I was struck, and very much gratified, when attending the Dental Convention, last year, at Asbury Park, to see the intense interest and appreciation with which the papers discussing electrical problems were received.

I read not long ago about some electrical experiments upon floating organisms. If a galvanic current be passed through a bath containing paramecia in sufficient abundance, a curious sight is observed. When contact is made, the whole crowd of paramecia fall into order with their noses towards the cathode, and begin to swim towards it in converging curves, while, if the current be reversed, the crowd breaks up, all its units turn around, and begin to swim away, as if of one mind, from the new anode to the new cathode. The creatures are evidently more "comfortable" when swimming with the electric current than the reverse way. In another experiment, a number of tadpoles were put into a lantern bath. They began to move about very leisurely, and to jostle each other in all directions. On sending a current of electricity through the bath there was prodigious excitement. As Dr. Waller, who conducted the experiment, described it, "The tadpole community seems to have gone mad, a writhing mass is all that can be distinguished; but the disturbance does not take long to subside, and now all the tadpoles are fixed as if at attention, heads to anode,—viz., traversed by a current from head to tail, stroked down the right way." It appears to me that the dentists, like the tadpoles, have begun to find that it is wise to yield themselves to the current of electrical progress that has set in; it points their heads the right way, and means, in the long run, less trouble, better work, and, last but not least, relatively better pay.

One of the great drawbacks to the adoption of electricity in general dental practice is the cheap outfit and the poor installation work. Any man who can put up an electric bell now calls himself an electrician. If it were not for the inspection department of the Board of Underwriters there would be, from bad wiring, such a crop of fires in large cities as would render the lighting of the streets entirely unnecessary. But there are no such official restrictions and precautionary measures for the protection of the dentist. Beware of cheap outfits. Do not try to save a dollar or two in your battery plant; get the best that is in the market. Go for your motors and your dental equipments to the best houses, that spend money on experimentation instead of letting you pay for it directly, and that insure careful inspection and sound installation, and do not subject you to the curses of defective wires, poor plugs, and inferior tackle of every description. I know of an instance in New York where the dentist had the hard luck to be taken in hand by a cheap, so-called electrician. Before he got through he spent five hundred dollars in his electrical outfit, and then he threw it up and vowed he would not have anything more to do with electricity, as it was a real fraud. Therefore, I say, dental electric outfits are like bicycles; if you want one to wear well, run smoothly, and save lurid language, get the best you can lay your hands on; you will get gratifying results, and you will not have any hard things to say about electricity.

Now, as to the electrical possibilities of the future in dentistry. Although we cannot gauge them, we have been lately getting some glimmering as to the trend they are likely to take. I wish I could help saying something about cataphoresis, but I cannot. Dr. Morton and Dr. Gillett have, I suspect, so worked you up to a keen edge on this subject of cataphoric work that the mere mention of that somewhat clumsy word may have the same effect on some of you that "rats" has on a skye-terrier, or, as they say, that "atrocities" has on Gladstone. I am trying to steer as clear as I can of all technicalities, either dental or electrical; but, if I have to talk "shop" for a minute or two, I trust you will forgive me. The coming work in cataphoresis will not only be vast in extent, but marvellous in its general bearing on dental practice. Many occasions constantly arise where anæsthetic drugs may be introduced into tissue and into dentine, and also other drugs, such as iodine, etc., may be used in the same way, and their familiar topical effects be vastly enhanced by the aid of electricity. It is impossible to define the limitations of this great aid to the local application of drugs within the cavity of the mouth. Broadly, it may be said that any drug that has been previously used without electricity within the mouth to produce specific effect may now be electrically used with tenfold its former power for good. The subject of cataphoresis is already very wide, and I am not going to enter further upon it. I would, however, say, as it leads up to another point, that the question of the resistance of the liquid used has a most important bearing in the therapeutical applications of cataphoresis. If the resistance is too high, as with chloroform, sulphuric ether, alcohol, glycerin, etc., little or no current is conveyed, and no cataphoresis takes place, while on the other hand, if the electric resistance is too low, as with strong saline and acid solutions, much current passes, but little or no cataphoric action takes place. Now, you are all familiar with the points of the late discussion on guaiacocaine. It may be, and undoubtedly is, an excellent anæsthetic, but it is ill-smelling and escharotic, and none of you like it. It has been proposed to add glycerin to it, but Mr. Wheeler will tell you that he does not like *that.* If the resistance is thus increased, more current will have to be used, and Mr. Wheeler, I am sure, does not want to spoil the record of his volt-selector, which is destined to be one of the standard instruments of the dental office. Dr. Morton, however, has found a way out of the difficulty. He says that guaiacol alone, and other similar substances and derivatives, in themselves non-conductors of electricity, by the addition of a very minute quantity of some innocent substance of an electrolytic nature, like salts of caffeine or quinine, or any salts of the alkaloids, may be caused to penetrate tissue by the aid of electricity, and thus exhibit anæsthetic effects unobtainable without the aid of the added electrolyte. But this is one of the points that you gentlemen will have to straighten out before the best results from cataphoresis can be reached.

Further developments in the uses of the different kinds of currents is another subject which is too comprehensive to be discussed here. I may, however, promise that the sinusoidal current will sooner or later find its way into dental laboratories. As you know, a dynamo-current is uneven, but the sinusoidal, with its very high vibrations, has soft, wave-like impulses; the current is agreeable and soothing to the patient, and more horse-power of electricity can be administered than if the frequency were lower and the wave less symmetrical. For example, the present sinusoidal machines run up to two thousand alternations per second, whereas the alternations per second of an induction coil would be, probably, at their highest limit, five hundred per second. Added to that, the graphic curve from the induction coil would be comparatively unsymmetrical,—*i.e.*, irregular. It is these two elements, the frequency and the graphic curve, or, as D'Arsonval calls it, "the characteristic of excitation," which determine whether the current is painful or not, and it is pain only which limits the extent to which the induced currents from ordinary medical apparatus may be administered. On this hand, I may mention, that guesswork in electrical dosage will soon be a thing of the past. The time is approaching when electricity will be prescribed and administered not only in measured units of intensity, density, and time, but with definite ideas of the electromotive force-curve, and of the therapeutic indications that a given curve may be expected to fulfil.

There are many other branches of electro-dental work in which we are on the verge of epoch-making improvements, and among them are the important ones of transillumination, implantation, and sterilization. For the purposes of transillumination, it is probable that more attention will be paid to the intensifying of the light. For instance, instead of confining the lamps for that purpose to small candle-powers, they might be run up to, say, twenty candle-power. Until lately, the difficulty has been that candle-power produced heat. This has been practically overcome by inclosing the first bulb in a second bulb through which water is made to flow. But here comes the fascinating question of the part which the phosphorescent or "etheric" light, the "Tesla glow," or, as it is also called, "the light of the future," will play in transillumination. I had the pleasure, about two years ago, of seeing in Mr. Tesla's laboratory a demonstration of the then newly-developed phosphorescent light. The room was darkened, and electrostatic currents played into it at the rate of possibly a million vibrations a second. Vacuum tubes, in fancy shapes, lay all around, and as these were taken up and lifted into the "field," they burst forth into beautiful luminescence, and the room was illuminated. Tesla then prophesied that it would not be long before our houses were lighted without wires, and even without lamps, and that the night would be made as day by means of phosphorescent light caused by the play of current through air-exhausted tubes. Last year a photograph of Mr. Tesla was taken by this light. As a photograph it was not much to boast of, but as a piece of history, it was most interesting. It was the first time that a photograph had been taken by that light. That line of work was taken up by a man in your own city, Mr. McFarlan Moore, who in the short time that has since elapsed has so developed it that he published a week or two ago a bold and striking picture, possessing the clearness and strength of a daylight photograph, which was secured by a three-minute exposure under the new light in his laboratory. If such strides can be made in twelve months, and we are coming at such a rate nearer to the electrician's and, for the matter of that, the dentist's ideal of light without heat, I think it may pay the dentists to let their spirit of investigation drift in the direction of transillumination by phosphorescent light.

Electricity, as an agent in implantation, etc., of teeth is a very attractive subject, and we are on the eve of great developments in this field ; but I must not detain you longer.

There is, however, an incident, of which I should like to tell you, which bears on another important branch of work,—sterilization. Some time ago a German physician wrote to Mr. Tesla and said that he had been experimenting with the play of etheric light on the bacilli of tuberculosis, and not only had he killed the microbes while experimenting, but he hoped by the same means to cure tubercular developments in the human subject. This letter, sad to say, was burnt up in the fire at the laboratory, and Mr. Tesla has never been able to find where it came from. Now, gentlemen, you will see how beautifully this bears on the question of sterilization: pyorrhœa alveolaris should have no further terrors when it can be swept out of the human jaw with a brush of glowing phosphorescence, and a new charm will be given to dental work when light will not only illumine, but purify and anæsthetize, and even kill the germs that work havoc within the mouth.

And now, the Röntgen rays,—no speech to-day is complete without something about them. I do not think any of us yet realize what they may mean to dentistry. If it can be established that the cathodic rays will, like light, kill microbes, you see at once where it carries you to. There is one thing, however, that cathodic rays have done that you gentlemen, I am sure, will take careful note of. They have passed through ivory and photographed pieces of metal on the other side. Where roots and other parts of the teeth are being treated, it is often necessary to know which teeth already contain fillings. There is now no further difficulty about this. As metallic substances intercept light to a greater degree than bone does, a cathodograph will show the location of all the fillings in the teeth which have already been operated upon. Dr. W. J. Morton has proposed to put the Röntgen rays within the cavity of the mouth, and a sensitive plate within the plate-holder outside on the cheek, and thus reproduce the teeth *in situ*, roots, fillings, etc. In this connection, it would also be possible to determine the existence of osteitis, deformations, pus-deposits, etc., in fact, the whole concealed pathology of the teeth in their relation to the jaw and gums would be thus revealed.

In conclusion, Mr. President and gentlemen, I would say, This subject is to me an intensely interesting one, and I shall watch keenly the new developments of electricity in dentistry. But among the names of those who will make their mark in this field, I shall look with very earnest expectation for some of the members of the Central Dental Association of Northern New Jersey, and I am confident that I shall not look in vain.

